# Self-control depletion is more than motivational switch from work to fun: the indispensable role of cognitive adaptation

**DOI:** 10.3389/fpsyg.2014.00933

**Published:** 2014-08-25

**Authors:** Junhua Dang, Shanshan Xiao, Siegfried Dewitte

**Affiliations:** ^1^Department of Psychology, Lund UniversityLund, Sweden; ^2^Department of Psychology, Peking UniversityBeijing, China; ^3^Research Center for Marketing and Consumer Science, Faculty of Business and Economics, Katholieke Universiteit LeuvenLeuven, Belgium

**Keywords:** self-control, resource depletion, cognitive control, switch costs, adaptation

It has been consistently demonstrated that people tend to perform more poorly on subsequent self-control tasks after completing an initial task that requires them to exert self-control (Hagger et al., 2010; Hagger and Chatzisarantis, 2014). The predominant explanation of such depletion effect claims that self-control taxes a limited resource that becomes drained with use (Muraven and Baumeister, 2000). Inzlicht and colleagues recently challenged the resource model by questioning the necessity and sufficiency of the resource metaphor for explaining self-control (Inzlicht et al., 2014). Instead, they presented a non-resource based process model. According to this model, self-control failure due to initial exertion is less about resource depletion but more about the motivated switching of task priorities from “have-to” and labor goals to “want-to” and leisure goals. We applaud such advance as the new account not only is evolutionarily and biologically more plausible but also can accommodate recent findings that are incompatible with the resource model (Inzlicht and Schmeichel, 2012; Inzlicht et al., 2014). However, we argue that the motivation-shift mechanism emphasized by the process model alone is not sufficient for explaining self-control depletion. A parallel cognitive adaptation mechanism must also be taken into account.

From a cognitive control perspective, the depletion effect is nothing mysterious but can be considered as a phenomenon similar to “switch costs” (Kiesel et al., 2010). The cognitive system is evolved to be able to actively adapt to given demands and buffer against situational changes. However, the inevitable cost is a reduced flexibility to promptly switch to a new demand. In a situation requiring consecutive exertion of effort, the control processes being recruited to adapt to the first self-control task would linger and hinder adaptation to the subsequent self-control task that requires different control processes (Botvinick et al., 2001; Dewitte et al., 2009). For example, if a dieting person is asked to control intake of palatable but unhealthy food after having performed emotion regulation, the recruitment of control processes for resisting temptation would be impeded as the control system is still geared toward regulating emotions.

The first implication of this reconceptualization is that engaging in a first self-control task could facilitate, rather than impair, self-control success in the second task when these two tasks require similar control processes because the control processes needed in the second task are already activated, as both experimental studies (Dewitte et al., 2009) and ecological momentary assessment (EMA) studies (O'Connell et al., 2008) have attested.

The second implication is that even if the control processes on which the two consecutive tasks rely are different, allowing respondents sufficient time to adapt to the task demands would cancel the depletion effect. Consistent with this implication, recent research showed that adapting to either the first task (Dang et al., 2013) or the second task (Barutchu et al., 2013) removed the depletion effect without rest or additional motivation. Further, there was also evidence showing a negative correlation between the adaptation level and the depletion effect even when the time for adaptation was limited, such that the more respondents adapted to the first task, the less errors they made on the second task (Dang et al., 2013).

The third implication suggests factors that can reduce switch costs would help to overcome self-control depletion. It has been demonstrated that positive affect, which could enhance flexibility of switching to new cognitive sets by directing attention to novel information (Dreisbach and Goschke, 2004), successfully neutralized the depletion effect (Wenzel et al., 2013). In the meantime, preparation is also critical for attenuating switch cost (Kiesel et al., 2010). Studies have shown that adapting to a series of self-control tasks could counterintuitively offset the depletion effect because the requirement of continuous exertion would help respondents get more prepared for switching to the following demanding task (Converse and Deshon, 2009; Xiao et al., in press).

Since these findings can hardly be reconciled with the motivation-shift mechanism, we argue that the cognitive adaptation mechanism addresses another unique feature of self-control depletion and parallels the motivation-shift mechanism. Finding out how these two processes interact with each other during consecutive exertion is an important question for future research. We suggest both the interference of the lingered control processes after initial exertion and the decreased motivation to engage in further effortful work contribute to impaired performance on the subsequent task. At the same time, there is also a competition between the adaptation process and the motivational process such that successful adaptation, either to the initial or to the following task, would gradually reduce the role of motivation-shift as adaptation attenuates the aversiveness of effort exertion that necessitates the motivated switching of task priorities (Inzlicht et al., 2014), thus helping to overcome self-control depletion without recurring to additional motivation.

## Conflict of interest statement

The authors declare that the research was conducted in the absence of any commercial or financial relationships that could be construed as a potential conflict of interest.
